# Effect of Fibroblast Growth Factor (FGF) 19 and 21 on Hip Geometry and Strength in Post-menopausal Osteoporosis (PMO)

**DOI:** 10.1007/s00223-024-01284-3

**Published:** 2024-09-28

**Authors:** EunJi Kim, Amelia. E. Moore, Dwight Dulnoan, Geeta Hampson

**Affiliations:** 1 Department of Chemical Pathology/Metabolic Medicine, North Wing, St Thomas’ Hospital, Lambeth Palace Road, London, SE1 7EH UK; 2 Osteoporosis Unit, Guy’s Hospital, London, UK; 3 Department of Endocrinology, Metabolic Bone Clinic, St Thomas’ Hospital, London, UK

**Keywords:** FGF19, FGF21, Osteoporosis, Hip geometry

## Abstract

Fibroblast Growth Factor (FGF) receptor signalling is important for skeletal development. The FGF19 subfamily which includes FGF19 and FGF21 are involved in bone metabolism, although their effects on bone mineral density (BMD) and bone strength remain unclear. To further characterise the influence of these two factors on the skeleton, we studied the association between circulating concentrations of FGF19 and 21 with BMD and parameters of hip geometry and strength in post-menopausal osteoporosis (PMO). The study cohort consisted of 374 women aged (mean [SD]) 68.7[12.3] years with PMO. FGF19 and FGF21 were measured in serum by ELISA. BMD was measured at the lumbar spine (LS), total hip (TH) and femoral neck (FN) (*n* = 277) by dual energy X-ray absorptiometry (DXA) and hip structural analysis (HSA) parameters (*n* = 263) at the narrow neck of the femur (NN), Intertrochanter (IT) and Femoral shaft (FS) were derived from DXA scans. FGF19 and 21 were not associated with prevalent fractures or BMD when corrected for covariates; age, BMI, smoking habits and alcohol intake. Log-transformed FGF 21 was negatively associated with HSA parameters including Outer Diameter (OD) (*p* = 0.019), Cross-sectional area (CSA) (*p* = 0.01), cross-sectional moment of inertia (CSMI) (*p* = 0.011), Section modulus (Z) (*p* = 0.002) and cortical thickness (Co Th) (*p* = 0.026) at the IT only. CSA, CSMI, Z and Co Th were significantly lower (*p* < 0.05) in women with FGF21 concentrations greater than the median (> 103.5 pg/ml). Our data suggest that FGF 21 may have potentially adverse effects on the skeleton. Further characterisation is needed, particularly as FGF 21 analogues or agonists may be used to treat obesity-related metabolic disorders.

## Introduction

The family of fibroblast growth factors (FGF) consists of a large number of proteins which play significant roles in organogenesis, development including skeletal development and metabolism [1]. The FGF19 subclass, referred to as the endocrine FGFs because they are present in the circulation, includes FGF19, FGF21 and FGF23. FGF23, produced by bone cells, is known for its effects on phosphate and vitamin D homeostasis [2].

FGF19, produced by ileal enterocytes, inhibits bile acid synthesis but also increases energy expenditure, glucose uptake in tissues and decreases hepatic glucose production resulting in increased insulin sensitivity [3]. FGF21, produced by the liver, has similar effects to FGF19 on glucose metabolism but it also decreases hepatic triglycerides and promotes fatty acid oxidation [4]. FGF19 has been shown to be inversely associated with body mass index, metabolic syndrome and Type 2 diabetes mellitus (DM) and levels increase following bariatric surgery [5, 6]. In contrast, FGF21 level is positively associated with metabolic conditions such as type 2 diabetes, insulin resistance and hsCRP, which is probably an adaptive response. Thus, FGF21 can be used as a predictive marker for type 2 diabetes mellitus [4, 7]. Studies have shown that exogenous administration of FGF21 in mice resulted in decreased blood glucose, lipids and improved insulin sensitivity and energy expenditure, suggesting that FGF 21 could also be a potential treatment in type 2 diabetes mellitus, obesity and disorders associated with the metabolic syndrome such as non alcoholic steatohepatitis (NASH) [8]. Over recent years, several clinical trials of human FGF21 analogues have been conducted in subjects with type 2 diabetes mellitus, obesity and fatty liver. Some of these trials showed improvement in lipid homeostasis and insulin sensitivity. Disappointingly, there were no effects on body weight reduction, although pegozafermin, an FGF21 analogue showed improvement in liver fibrosis in patients with NASH [9]. ldafermin, a FGF19 analogue, has also been shown to reduce liver fat and demonstrated a trend in improving hepatic fibrosis in a phase 2 trial [10].

Several members of the FGF family have been shown to play important roles in bone growth, skeletal development and in the pathogenesis of bone-related diseases [11]. However, the role and mechanism of action of FGF19 and 21 on the skeleton is less well studied. Studies on the chromosomal location of FGF19 and its expression in cartilage suggest a role for FGF19 in bone metabolism, although its specific function is less clear [12]. In animal and in-vitro studies FGF19 was shown to enhance osteogenic differentiation in obese mice fed a high-fat diet, demonstrating protection against obesity-induced bone loss [13]. In clinical studies, circulating concentration of FGF19 has been shown to be positively correlated with bone mineral density (BMD) in post-menopausal women [14].

FGF21 is also thought to regulate bone metabolism. FGF21 gain of function reduces bone mass by inhibiting osteoblast formation, stimulating adipogenesis instead from bone marrow mesenchymal stem cells [15]. Data from clinical studies have, however, had mixed results. A positive correlation between FGF21 concentrations and BMD in healthy women has been shown [16]. Other studies have not demonstrated any association between FGF 21 and BMD [17], although a significant negative association was seen with trabecular bone score (TBS) in post-menopausal women [18].

There is a lack of studies investigating the effect of FGF19 and FGF21 on parameters of bone quality and strength in the clinical setting. Understanding the effects of FGF19 and FGF21 on bone metabolism is important as FGF19 and FGF21 analogues may be used to treat disorders associated with the metabolic syndrome in future. We hypothesised that FGF19 and FGF21 may be associated with decreased bone strength. To further elucidate the effects of FGF19 and 21 on bone quality and strength, we conducted a study investigating the relationship between these 2 factors with BMD and parameters of hip geometry and mechanical strength in post-menopausal osteoporosis (PMO).

## Material and Methods

### Subjects

We studied 374 community-dwelling ambulant post-menopausal women aged (mean [SD]) 68.7[12.3] years with post-menopausal osteoporosis (PMO) on treatment with oral bisphosphonates with a mean duration of treatment of 3.6 [3.3] years as previously described [19]. Three hundred and fifteen women (84%) were on alendronate and 59 (16%) were taking risedronate. Two hundred and fifteen (67%) women had a history of one or more fractures which included wrist fractures (*n* = 83), peripheral fractures (pelvis, tibia, fibula, humerus, ankle, patella, metatarsals) (*n* = 171), hip fractures (*n* = 12) and vertebral fractures (*n* = 30) as previously described [19]. They were recruited from the metabolic bone clinics at Guy’s Hospital (*n* = 202), the osteoporosis unit (*n* = 71), through community advertising (*n* = 87) and primary care (*n* = 14) for  into a study looking at the effect of vitamin K status on fracture risk and hip strength. At initial attendance to the osteoporosis unit, a questionnaire was applied which provided information on their demographics (age, BMI), lifestyle factors (smoking habits, alcohol intake), medical and drug history including previous fractures and length of treatment with bisphosphonates. Participants with secondary osteoporosis and on other bone-modifying drugs were excluded.

Routine laboratory test results were available on 267 (71.4%) women. BMD data obtained from their most recent DXA scans (within 12–18 months) of their enrolment in the initial study was available on 277 (74%) women. This is shown in Table 1.

**Table 1:** Summary of baseline characteristics of study participants

Parameters (*n* = 374)	Mean [SD]
Age at screening (years)	68.7 [12.3]
Body mass index (BMI) (kg/m^2^)	23.2 [3.9]
Current or Ex-smoker, n (%)	18 (4.8%)
Alcohol consumption > 3 units/day, n (%)	9 (2.45%)
Prevalent fracture n (%)	251 (67%)
Length of treatment with oral bisphosphonate (years)	3.6 [3.3]
*Bone mineral density (BMD) (n = 277)*	
BMD at the lumbar spine (g/cm^2^)	0.793 [0.101]
T -score Lumbar spine	− 2.3 (0 .92]
BMD at the Total Hip (g/cm^2^)	0.747 [0.091]
T -score Total Hip	− 1.56 [0 .81]
BMD at the Femoral Neck (g/cm^2^)	0.627 [0.09]
T-score Femoral neck	− 2.0 [0 .81]
*Biochemical Parameters (n = 267)*	
Estimated GFR (ml/min)	79 [17]
Serum albumin (g/L)	46 [2.7]
Serum creatinine (umol/L)	67 [12.8]
Albumin adjusted calcium (mmol/L)	2.37 [0.1]
Parathyroid hormone (PTH) (ng/L)	38 [14]
25(OH)vitamin D (nmol/L)	77 [25]

### Dual Energy X-ray Absorptiometry (DXA) and Hip Structure Analysis (HSA)

BMD was measured by DXA at the lumbar spine (LS), total hip (TH) and femoral neck (FN) using the Hologic Discovery scanner (Hologic, Inc. Bedford, MA). The CV for BMD was 1.0 and 1.2%, at the LS and TH respectively.

Hip geometry analysis was performed using the HSA program using the femur image within the DXA analysis software [20]. The regions of interest defined by the HSA software included 3 hip sites; the narrow neck (NN) defined as the narrowest diameter of the femoral neck, the intertrochanter (IT) along the bisector of the neck shaft angle and the femoral shaft (FS) which is 2 cm distal to the midpoint of the lesser trochanter. The analyses were done by a certified DXA technologists in accordance with the standardised HSA analysis protocol. The program provided the following measurements at each site; [1] subperiosteal (outer) width or diameter (OD) [2] endocortical (inner) width or diameter (ED) [3] cross-sectional area (CSA) which provides an index of resistance to axial forces, [4] estimated cortical thickness (Co Th), [5] cross-sectional moment of inertia (CSMI) which gives an estimate of resistance to bending forces and structural rigidity, [6] section modulus (Z) which is an indicator of bending strength, [7] buckling ratio (BR) which provides an estimate of susceptibility to local cortical buckling under compressive loads, [8] neck shaft angle (NSA) and [9] hip axis length (HAL).

### Biochemical Assessment

Routine laboratory measurements were carried out and included renal/liver/bone profile, parathyroid hormone (PTH). They were done by standard laboratory methods on the Roche automated analysers (Roche Diagnostics Limited, West Sussex, UK). 25-hydroxy vitamin D (25(OH)D) was measured using an immunoassay on the automated Abbott Architect analyser (Abbott Laboratories, Abbott Park, Illinois, USA). PTH assay CVs were < 5% at PTH concentrations of 41 and 105 ng/L. 25(OH)vitamin D assay CVs ranged between 5.0 and 10.7% at serum 25 (OH)vitamin D concentrations between 25 and 85 nmol/L. The reference ranges for PTH were 10–65 ng/L, serum albumin: 40–52 g/L, serum creatinine: 45-84 µmol/L. Serum 25(OH)vitamin D > 50 nmol/L is considered sufficient, 25–50 nmol/L: insufficient and < 25 nmol/L: deficient.

FGF19 was measured using the human FGF19 DuoSet ELISA (DY969) and ancillary reagent kit (DY008) (Biotechne Ltd, Abingdon OX14 3NB, UK). Following optimisation, the detection limit (LOD) was 6.55 pg/ml and the limit of quantification (LOQ) was 21.6 pg/ml. The inter-assay coefficient of variation (CV) was 9.1% and the intra-assay CV was 5.0%. FGF19 was measured in serum aliquots which had not been thawed previously. The human FGF21 Duoset (DY2539) from Biotechne was used to measure serum FGF21. The LOD and LOQ for FGF21 were 7.4 and 24.4 pg/ml respectively. Inter-assay CV and intra-assay CV were 10.2 and 8.2% respectively. There was no cross-reactivity between FGF19 and FGF21 ELISAs. Serum FGF19 and FGF21 were measured on samples from 358 women.

### Statistical Analyses

Statistical analysis was performed using IBM SPSS Statistics version 26 for Windows (https://www.ibm.com/products/spss-statistics). Mean and standard deviation (SD) or median (interquartile range; IQR) were estimated for all continuous variables. Non-parametric data including serum FGF19 and FGF21 were log-transformed. Mann–Whitney *U* test was used to compare FGF19 and FGF21 between women with prevalent clinical fractures and those who were fracture-free. Spearman rank correlation was used to assess the correlation between FGF19 and FGF21 with biochemical parameters. To determine the association between serum FGF19 and FGF21 and fractures, we derived odds ratios (ORs) by binary logistic regression analyses, adjusting for possible confounders such as age, BMI, lifestyle risk factors such as smoking habits and alcohol intake. Pearson’s correlation was used to assess the correlation between BMD and Log-transformed FGF19 and FGF21. Multiple linear regression analysis was used to investigate any association between BMD at the LS, FN and TH, HSA parameters with FGF19 and 21 after correction for confounders which included age, BMI, lifestyle factors and duration of treatment with bisphosphonates. We compared HSA parameters between women with FGF19 and FGF21 below and above the median value for each factor using Mann–Whitney *U* Test. A two-sided *‘p’* value of < 0.05 (95% confidence interval) was accepted as statistically significant.

## Results

### Serum FGF19 and FGF21 Concentrations in the Study Population

**Fig. 1:**
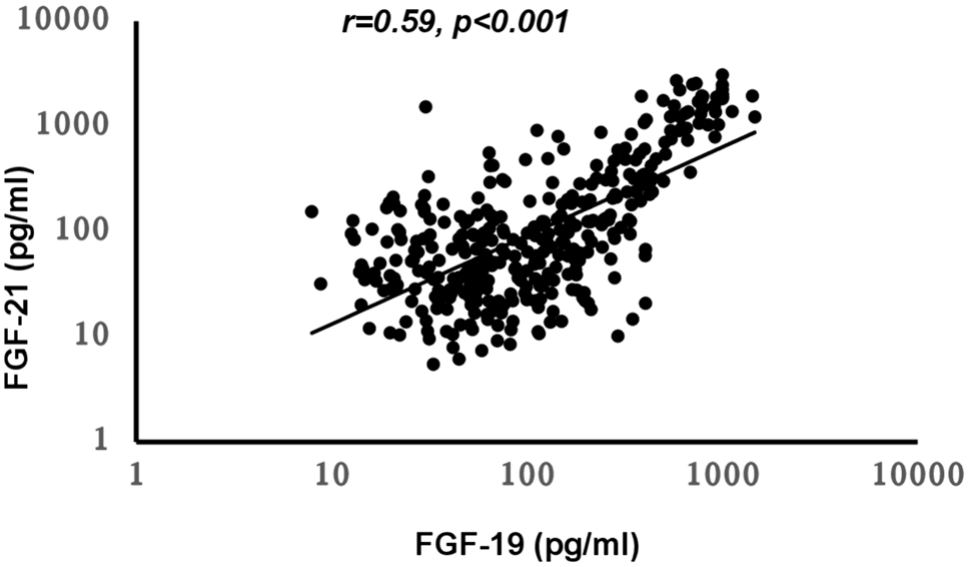
Relationship between serum FGF 19 and FGF21 concentrations (r = 0.59, *p* < *0.001*)

The median (IQR) FGF19 and FGF21 were 103.5 (221) pg/ml and 81(196.6 pg/ml) respectively. There was a significant correlation between FGF19 and 21 (r = 0.59, *p* < 0.001) as shown in Fig. 1. There were no significant correlations between FGF19 and FGF21 with age, BMI and other biochemical parameters; eGFR, PTH and 25 (OH)vitamin D, although we observed a small but significant correlation between FGF21 and albumin-adjusted calcium (r = 0.12, *p* = 0.05). Because of the potential association between FGF19 and FGF21 levels with bone metabolism and bone turnover, we investigated the relationship between these 2 factors and duration of treatment with bisphosphonate which impacts bone metabolism. There was a significant correlation between serum FGF19 and FGF21 with length of treatment with bisphosphonates (FGF19; r = 0.112, *p* = 0.035, FGF21; r = 0.131, *p* = 0.014), although this was no longer significant when corrected for age, BMI, alcohol intake and smoking habits in a multilinear regression model. Smoking is an important risk factor for cardiovascular disease and as FGF19 has also been shown to be associated with cardiovascular risk factors, we tested any association between FGF19 levels and smoking habits. Following multilinear regression analysis after correction for confounders such as age, BMI and biochemical parameters, a significant negative association was seen between FGF19 and smoking habits (beta − 0.14, *p* = 0.033). FGF19 tended to be higher in non-smokers compared to smokers (FGF19: median (IQR), non-smokers; 109(227), smokers; 63(116) pg/ml, *p* = 0.1).

### Association Between Serum FGF19 and FGF21 and Fracture Risk

There were no significant differences in serum FGF19 and FGF21 in women who had sustained a previous fragility fracture compared to those who had not (FGF19 median (IQR) no fracture: 132.3 (276.4) pg/ml, previous fractures: 98.1 (169) pg/ml, *p* = 0.093, FGF21 median (IQR) no fracture: 100.7 (258)pg/ml, previous fractures: 74.3 (178.2) pg/ml, *p* = 0.115). There was no significant association between FGF19 and FGF21 and fracture risk following logistic regression analyses with adjustment for confounders including age, BMI, length of treatment with bisphosphonates, 25(OH)vitamin D and lifestyle factors (FGF19; OR: 0.601 (CI 0.34–1.075) *p* = 0.086, FGF21; OR: 0.791 (CI 0.0.5–1.26) *p* = 0.32).

### Association Between Serum FGF19 and FGF21 with BMD and HSA

BMD and HSA data were available in 277 and 263 participants respectively. There was a small but significant correlation between log-transformed FGF19 and BMD at the TH only (r =  − 0.12, *p* = 0.045). However, following multiple regression analyses, we did not observe any significant association between serum FGF19 and FGF21 concentration with BMD at the LS, FN and TH. In multiple linear regression analyses, there was no significant association between log-transformed FGF19 and any of the HSA parameters at the NN, IT and FS. There were significant negative associations between log FGF21 and HSA parameters including OD (*p* = 0.019), CSA (*p* = 0.01), CSMI (*p* = 0.011), Section modulus (Z) (*p* = 0.002) and Co Th (*p* = 0.026) at the IT only (Table 2).

**Table 2:** Association between the HSA parameters at the intertrochanter (IT) (dependent variable) and Log-transformed FGF21 serum concentrations

Hip Structural Analysis (HSA) Parameters Intertrochanteric region (IT)	Log FGF21 concentrations	
	β-coefficient	‘*p*’ *value*
Outer Diameter (OD)	− 0.153	*0.019*
Cross sectional area (CSA)	− 0.161	*0.01*
Cross sectional moment of inertia (CSMI)	− 0.165	*0.002*
Section modulus (Z)	− 0.196	< *0.001*
Cortical Thickness (Co Th)	− 0.145	*0.026*
Endocortical Diameter (ED)	− 0.071	*0.28*
Buckling Ratio (BR)	0.079	*0.22*

The multilinear regression model included correction for variables such as age, BMI, lifestyle factors (smoking, alcohol intake), length of treatment with oral bisphosphonate. *p* < *0.05* is considered significant

**Fig. 2:**
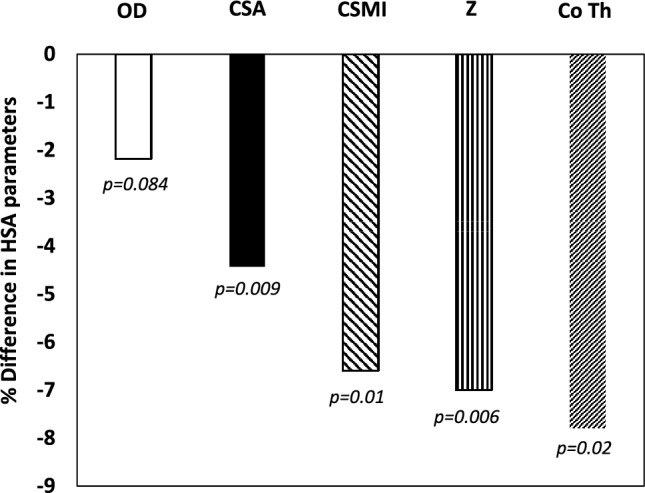
% difference in HSA parameters at the intertrochanteric (IT) area based on the median value of serum FGF21 concentrations (103.5 pg/ml) group with FGF21 concentrations above the median compared to those below the median (103.5 pg/ml). OD: outer diameter, CSA: cross-sectional area, CSMI: cross-sectional moment of inertia, Z: section modulus, Co Th: cortical thickness

There were significant differences in the HSA parameters at the IT including OD, CSA, CSMI, Z and Co Th when the study population was divided into two groups based on the median of FGF21 (103.5 pg/ml) (Table 3). Those with FGF21 concentrations above the median value tended to have significantly lower OD, CSA, CSMI, Z and Co Th at the IT. There were no significant differences in age, BMI, BMD between the 2 groups. Figure 2 shows the % difference in HSA parameters at the IT in those with FGF21 concentrations above the median compared to the group with FGF21 lower than the median (103.5 pg/ml).

**Table 3:** Characteristics and HSA parameters at the intertrochanteric region (IT) between the 2 groups of participants based on serum FGF21 median value

Parameters Mean [SD]	Serum FGF21 < median value, < 103.5 pg/ml	Serum FGF21 > median value, > 103.5 pg/ml	*p* value
Age at screening (years)	68 [7.1]	68.3 [7.5]	0.39
Body mass index (BMI) (kg/m^2^)	23.2 [4]	23.2 [3.9]	0.8
BMD at the lumbar spine (g/cm^2^)	0.791 [0.096]	0.789 [0.10]	0.37
BMD at the Total Hip (g/cm^2^)	0.752 [0.084]	0.741 [0.099]	0.33
BMD at the Femoral Neck (g/cm^2^)	0.635 [0.087]	0.619 [0.096]	0.74
Prevalent Fractures n (%)	126 [70%]	116 [65%]	0.31
*Hip Structural Analysis (HSA) Parameters* *Intertrochanteric region (IT)* *Mean [SEM]*			
Outer Diameter (OD) (cm)	5.490[.039]	5.370[.0414]	0.084
Cross sectional area (CSA) (cm^2^)	4.060[.056]	3.880[.057]	0.009
Cross sectional moment of inertia (CSMI) (cm^4^)	11.240[.27]	10.50[0.33]	0.01
Section modulus (Z) (cm^3^)	3.530[.072]	3.28[0.064]	0.006
Cortical Thickness (Co Th) (cm)	0.335[0.014]	0.309[0.005]	0.02
Endocortical Diameter (ED) (cm)	4.82[0.047]	4.75[0.043]	0.2
Buckling Ratio (BR)	10.04[0.18]	10.46[0.20]	0.3

## Discussion

This study found significant negative associations between circulating FGF21 and parameters of hip geometry and strength at the IT. There were significant differences in several HSA parameters between subjects with FGF21 concentrations above the median value compared to those below the median value. We did not observe any associations between the bone parameters and circulating FGF19. Our data suggest that FGF21 may exert a negative effect on bone structure and strength, particularly at the hip.

FGF19 and FGF21, members of the FGF19 subclass, act via FGFR receptors in the presence of the transmembrane coreceptor beta-klotho (KLB). Although there is an overlap in their insulin-sensitising effects, they are regulated by different physiological and pathological factors [21]. A small negative association was seen between FGF19 and smoking habits which tended to be lower in smokers. Previous reports have shown negative associations between FGF19 and cardiovascular risk factors including triglycerides and the plasma atherosclerosis index [22]. Although smoking is an important cause of cardiovascular disease, the association between FGF19 and smoking has not previously been described. Further larger studies are needed for confirmation.

Members of the FGF19 subfamily have also been linked with bone metabolism although this remains unclear and is still the subject of debate [23]. Studies looking at the relationship between circulating FGF19 and FGF21 with BMD have been inconsistent as positive as well as negative correlations have been found, depending on the skeletal sites; lumbar spine or hip regions [14–16]. These differences have been attributed to differences in study design or population characteristics/demographics. We found a significant negative correlation between FGF19 only and BMD at the TH. This negative correlation has previously been reported in adults older than 60 years [24]. If there is an effect of FGF19 on bone metabolism, albeit a small one, this may involve the bile acid pathway as increased circulating FGF19 suppresses bile acid synthesis [25]. Recent studies have shown that bile acids may have a protective effect on bone [14]. However, we did not observe any significant associations between FGF19 with BMD at any skeletal sites when adjusted for confounders in agreement with other studies which also have not shown any significant relationship between FGF19 with BMD [17]. The lack of association seen in our study may also have been related to the use of bisphosphonates in our study population, although this was adjusted in our analyses. We did not find any association between FGF19 with fractures. One explanation for our findings is the small study numbers. The participants were also on bisphosphonates which may have affected fracture risk, although the reported fractures occurred prior to bisphosphonates. Although FGF21 has been shown to negatively impact bone formation and stimulate bone resorption [15], no association was seen in this study between FGF21 and fracture risk or BMD [17]. This may be, in part, due to similar factors detailed above such as the small study numbers and the use of bisphosphonates.

Although there have been several studies looking at the relationship between FGF19 and FGF21 with BMD, there is a paucity of information at the effect of these factors on bone micro-architecture and strength. DXA-derived hip structural analysis (HSA) has been shown to be useful in the evaluation of hip geometry and mechanical strength as these parameters are good fracture predictors, particularly hip fractures, independently of BMD [26]. Despite the lack of association with BMD, our data show that only FGF21 was negatively associated with HSA parameters at the IT. This suggests that hip strength parameters including measures of cortical area, structural rigidity and bending strength may be affected by FGF21. Subjects with serum FGF21 concentrations above the median value had lower OD, CSA, CSMI, Z and Co Th values at the IT. The percentage difference in those HSA parameters based on FGF21 concentrations was of similar magnitude to those observed in epidemiological studies when comparing subjects with hip fractures to those without [26]. There have been very few studies on FGF21 and bone structure and strength in humans, although a small study using high-resolution computed tomography (HR-pQCT), in women with anorexia nervosa (AN) showed that serum FGF21 concentrations in both women with (AN) and normal weight (NW) as controls were inversely associated with trabecular parameters at the radius and a positive association was seen with trabecular separation contributing to a decrease in bone strength at the radius [27]. These data are consistent with our findings, although our study population was older. The effect of FGF21 on predominantly trabecular micro-architecture at the radius would be in agreement with our observations at the IT as this region is composed mainly of trabecular bone. Other studies in post-menopausal women with impaired glucose (pre-diabetes) have also shown that FGF21 Levels were independently associated with trabecular bone score (TBS) [18]. Higher circulating concentrations of FGF21 have been observed in diabetes mellitus and may be involved in the adverse effects of diabetes mellitus on the skeleton and fracture risk [28, 29]. Further studies are needed to investigate this.

The mechanisms of action of FGF21 on bone cells remain unclear. Some studies have shown that the negative effects of FGF21 on bone may be related to its stimulation of adipogenesis of bone marrow stromal cells, inhibition of osteoblast activity and increased osteoclast activity via activation of peroxisome proliferator-activated receptor gamma (PPAR-γ) [15, 30]. Indeed, PPAR-γ agonists such as rosiglitazone used as an anti-diabetic medication have been shown to have adverse effects on the skeleton [31]. However, a more recent study found that FGF21 does not have any direct effects on the PPAR pathways in the regulation of bone metabolism [32]. Thus, further investigations are needed to elucidate the molecular mechanisms of FGF21 on bone, particularly as FGF21 analogues/mimetics for the treatment of obesity-related metabolic complications are in various phases of clinical trials.

Our study shows that circulating FGF21 concentrations are independently and inversely associated with parameters of hip geometry and mechanical strength suggesting that FGF21 may have an adverse impact on the skeleton. However, the study has some limitations. Causality cannot be inferred due to its cross-sectional design. The observed associations may have been affected by the presence of unknown confounding variable. Although we corrected for a number of known confounders, this did not include activity level. We did not observe any associations with the presence of fractures. This may be related to the small study numbers and the use of bisphosphonates, although we adjusted for the duration of treatment with bisphosphonates. We used DXA-derived images to determine the HSA parameters which include certain assumptions, although HSA parameters derived at the hip have been shown to correlate highly with high-resolution QCT [33].

In conclusion, this study shows that FGF21 may adversely affect hip strength in post-menopausal women. Further confirmatory studies are needed to fully evaluate the impact of FGF21 production on skeletal metabolism, particularly in high risk populations such as in type 2 Diabetes Mellitus where FGF21 concentrations are higher. The effect of FGF21analogues on bone health and fracture risk should also be investigated, prior to their use in treating obesity-related disorders.
